# Analysis of spine motion during prehospital extrication procedures in motorsport

**DOI:** 10.1007/s00068-024-02608-6

**Published:** 2024-08-07

**Authors:** Davut Deniz Uzun, Roman Klein, Adrian Rittmann, David Häske, Niko R. E. Schneider, Michael Kreinest

**Affiliations:** 1 University Heidelberg, Medical Faculty Heidelberg, Department of Anesthesiology, Heidelberg, Germany; 2 Surgical practice OC Orthochirurgie, Ludwigshafen, Germany; 3 BG Trauma Center Ludwigshafen, Ludwigshafen, Germany; 4 Center of Public Health and Health Services Research, University Hospital Tübingen, Tübingen, Germany; 5 Emergency Medical Service, German Red Cross, Reutlingen, Germany; 6 Department of Anesthesiology, Giessen University Hospital, Giessen, Germany

**Keywords:** Immobilization, Spinal motion, Spine, Racetrack, Extrication procedures

## Abstract

**Purpose:**

The appropriate extrication techniques for trauma patients after car accidents remain a topic of controversy. Various techniques for immobilizing the cervical spine during prehospital extrication have been investigated.

**Methods:**

This explorative study compared the amount of spinal motion during five different extrication procedures from a racecar and a rallycar performed by two teams: a professional motorsport extrication team and a team of professional emergency medical technicians (EMTs). Two different microelectromechanical systems were used to measure spinal motion, and a motionscore was calculated to compare the amount of remaining spinal motion. A high motionscore indicates high remaining motion and a low motionscore indicates low remaining motion.

**Results:**

The use of an extricable seat results in a mean overall motion score of 1617 [95% CI 308–2926]. Emergency extrication without equipment resulted in the lowest overall motionscore 1448 [95% CI 1070–1826]. In case of urgent extrication the Extrication team attained a motionscore of 2118 [95% CI 517–3718] and the EMT team a motionscore of 2932 [95% CI 1427–4435]. When performing the procedure with the aid of a rescue boa, the EMT team achieved an overall mean motionscore in the same range 2725 [95% CI 568–4881] with boa vs. 2932 [95% CI 1427–4435] without boa. When mean scores of individual spinal segments were analyzed, we found that the EMT team did especially worse in immobilizing the cervical spine 198 vs. 758.

**Conclusions:**

Regular training of extrication procedures has paid off considerably in reducing spinal movement during extrication from a racecar. If an extricable seat is available, extrication should be performed using it. However, if emergency extrication is necessary, an additional manual cervical spine immobilization should be conducted using the Rautek maneuver to sufficiently reduce cervical spine movement.

## Introduction

### Development of rescue procedures in motorsports

Motorsports has seen many severe accidents that influenced development of rescue concepts of injured racing drivers (RDs). Notably, the flawed rescue maneuvers of Philippe Streiff and Emanuele Pirro (both 1989) made the Fédération Internationale de l’Automobile (FIA) invest in professionalization of racetrack emergency medical services. Stemming from blunt high-energy trauma, spinal injuries make up a significant fraction of injuries inflicted by racecar accidents [1]. The main areas for spinal cord injuries are the lower cervical spine and the upper and middle thoracic spine [2]. Motorsport has its different facets and technical peculiarities, on the one hand these consist in the organization and on the other hand in different vehicle technologies and vehicle types. For example, there are clear differences between Formula 1 (F1) and rally racing [1]. Rallying and off-road events pose unique challenges as there is no formal extrication team and often only one vehicle is available to assist two competitors. In such situations, it may take some time to obtain rescue or medical assistance. Competitors are permitted to remain in the vehicle until skilled assistance arrives, provided it is safe to do so. However, there is no formal extrication team, and multiple crews must collaborate under medical supervision. Rallying has specific techniques, such as the Rautek maneuver, which enable a single rescue worker to extract a driver in an emergency.

### Current recommendations for spinal immobilization

From 1989, a task force worked on guidelines on the extrication of injured RDs. So-called extrication teams (four to six members including one physician) are nowadays responsible for the extrication of injured drivers. All team members need to undergo special education and are provided with equipment that permits axially aligned spine-protecting rescue of the injured driver. The training for extrication seats used in F1 and other single-seater series has always been didactic and structured. However, as the issues to overcome have become more complex, it is sometimes necessary to think outside the box and make quick decisions based on experience. Therefore, it is best to have an enthusiastic regular team that trains and works together, rather than a team that is brought together for one event a year. The use of the rigid cervical collar is a controversial topic among first responders worldwide [3–8]. Many are moving away from the hard collar due to the lack of evidence supporting their use and the potential problems that can outweigh any benefits. This shift is acknowledged by many national guidelines [9]. It is important to note that there is a *significant difference* in the forces involved in motor sports compared to those in road cars. The safety systems in a race car, both passive and active, differ significantly from those found in a road car. It is not appropriate to directly compare the two, and translating the mechanism of injury from a road traffic collision to a motor racing accident is not sensible. Data from the motorsport sector is scarce, which presents a significant challenge. The above-mentioned guidelines are nowadays valid beyond the racetrack and have been incorporated in the guidelines of the German Society of trauma surgery [10]. Three modes of extrication are described (descending time spent on extrication):

1. **Elective extrication**: non-emergency situation with a patient in stable condition that allows to delay further treatment in order to perform a complete spine-protecting extrication.
2. **Urgent extrication**: intermediate situation with a patient in condition that allows a short delay of further treatment of only a few minutes in order to perform an inline extrication.
3. **Emergency extrication**: emergency situation with a patient in condition that permits no delay of further treatment, i.e. imminent cardiopulmonary resuscitation or a burning patient.

**Fig. 1:**
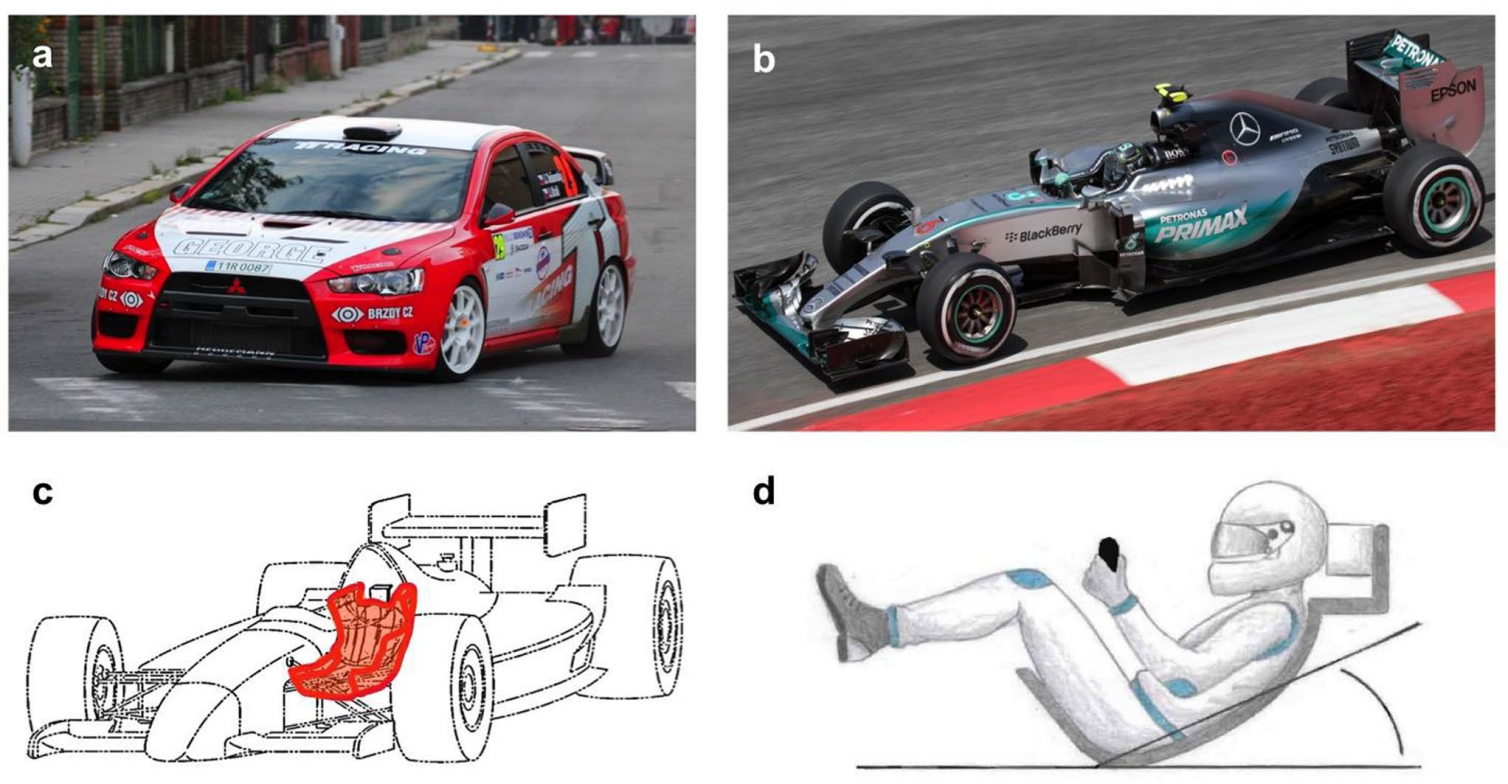
In contrast to a rallycar (**a**), a F1 car (**b**) is equipped with an extricable seat (**c**) that requires a special sitting position (**d**)

In some motorsports categories like F1, the use of an extricable seat is mandatory. This allows for the driver who is in an almost lying position (Fig. 1d) to be extricated with his seat as one following transfer to a spine board or vacuum mattress. Despite widespread consensus on the necessity of spinal immobilization, clinical trials with a high evidence level are lacking [11–13].

### Aims

The goal of this study was to compare (i) different extrication procedures and (ii) the influence of special extrication training with respect to remaining spinal motion during extrication procedures.

## Methods

For precise evaluation of the different extrication procedures, it was necessary to record movement of all spinal areas in a high temporal resolution. We used two different microelectromechanical systems for recording:

### Cervical spine (C1-C7)

**Fig. 2:**
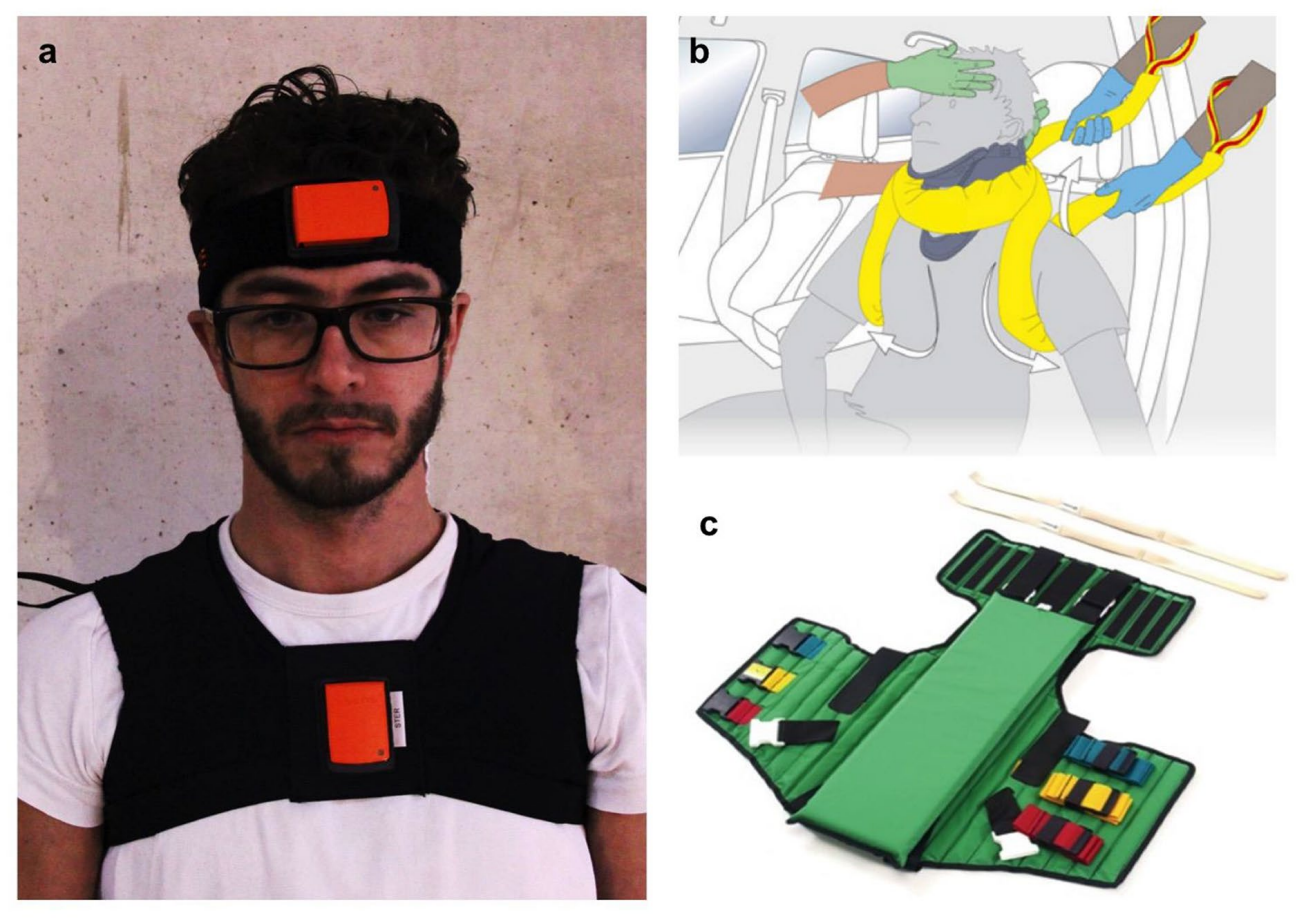
Xsens MT-system for recording cervical spine motion with the sensors attached to forehead and sternum of the RD (**a**). Schematic drawing of the extrication procedure using a rescue boa (**b**). The Kendrick Extrication Device (KED; **c**)

Two inertial measurement units (IMU) captured the movement of the cervical spine. We used the MT-system distributed by Xsens (Xsens Technologies, Netherlands, Fig. 2a). The IMUs were attached to the driver’s forehead and sternum. One unit consisted of three integrated accelerometers and gyroscopes each and allowed three-dimensional movement analysis with a resolution of 0.05° and a frequency of 50 Hz.

### Thoracic spine (Th1-Th9), thoraco-lumbar junction (Th10-L2) and lumbar spine (L3-L5)

A different system was utilized to capture the remaining spinal levels. We used the Epionics Spine System (Epionics Medical GmbH, Germany). It consisted of two strips applied to the paravertebral skin including two accelerometers as well as 24 strain gauge sensors. With this setup, three-dimensional spinal movements were recorded with a resolution of 0.05° and 50 Hz.

Covering the complete spine, this setup was the basis for recording of flexion/extension, rotation to the left/right side in each of the four spinal segments. Additionally, lateral bending to the left/right side was also analyzed in the cervical, thoracic and lumbar spine. The measurement of lateral bending in the thoracolumbar junction was technically impossible employing the above-mentioned sensors. An original measurement of the cervical spine is shown in Fig. 2a. The acquired data was then subjected to analysis by the motionscore as outlined below.

### Assessment of spinal movement

Spinal movement was assessed by the motionscore (MS). It is a dimensionless score to assess spinal movement in general and uses recorded data by microsensors to quantifies them in a score as described before [14–16]. Briefly, it is based on the following assumptions:

1. The amount of spinal movement that causes further damage to an injured spine is unknown.
2. As little spinal movement as possible is thought to be best.
3. If spinal movement occurs, small angular movement in a longer period is better than extensive angular movement in a shorter period of time [14].

These three assumptions are based on general physical and medical principles but should not be interpreted as hypotheses that have already been proven to be true. In contrast to analyzing a static range of motion that only determines maximum angulation and thus ignoring temporal resolution, the motionscore evaluates every partial movement relating to angulation and time [14–17]. The *lower* the motionscore, the *less* harmful the procedure.

### Extrication procedures

**Fig. 3:**
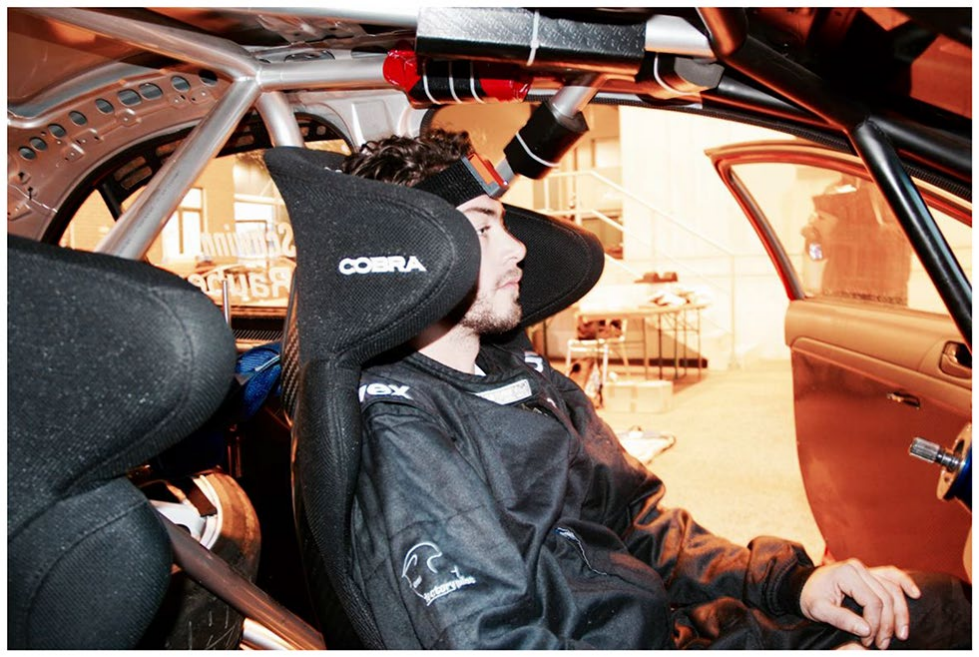
Driver sitting in the rallycar with the Xsens MT-system for recording cervical spine motion with the sensors attached to forehead

Spinal movement was evaluated during the extrication of two healthy male volunteers (volunteer 1: height 180 cm, body weight 77 kg, BMI 23,8; volunteer 2: height 175 cm, body weight 70 kg, BMI 22,9, Fig. 3) with comparable anatomy. Each of the following extrication procedure was performed three times by a professional Extrication Team according to the recommendations of the FIA medical commission [18, 19]. In addition, the rescue was supplemented by a so-called rescue boa (X-CEN-TEK, Wardenburg/Germany). The rescue boa is employed in the evacuation of patients or individuals from an environment that renders targeted work impractical or when time constraints necessitate it. Furthermore, the device is employed to optimally immobilize the cervical and thoracic spine areas in accordance with the circumstances under which it is being used, and in a manner that is consistent with the instructions for use (Fig. 2b). The so-called Rautek rescue maneuver was also used. The rescue maneuver is a measure for rescuing people from a danger zone. The centre of gravity position makes it easier to move people who are significantly heavier than the rescuer. During the rescue maneuver, the rescuer stands behind the seated casualty, reaches under their armpits on both sides and grasps the casualty’s forearm placed across their chest with both hands.

1. Urgent extrication from a rallycar (Fig. 1a) using cervical collar and spine board without a rescue boa: Manual immobilization of the cervical spine was performed during the complete procedure. A cervical collar was applied to the sitting RD. The belt system was then opened and the RD moved forward as far as necessary to rotate him axially and pull him on the provided spine board.
2. Urgent extrication from a rallycar (Fig. 1a) using a cervical collar, spine board with a rescue boa: Manual immobilization of the cervical spine was performed until a cervical collar was applied to the sitting RD. The rescue boa was put on the patient after the belt system was opened (Fig. 2b). The rescue boa handles were used to pull the RD onto the spine board.
3. Elective extrication from a rallycar (Fig. 1a) using cervical collar, Kendrick Extrication Device (KED; Fig. 2c) and spine board: Manual immobilization of the cervical spine was performed. A cervical collar was then applied to the sitting RD. The belt system was opened and the RD moved forward as far as necessary to apply the KED. Using the KED’s handles, the RD was pulled on the spine board.
4. Extrication from a F1 car (Fig. 1b) using the extricable seat (Fig. 1c): Manual immobilization of the cervical spine was performed until technical immobilization. After the steering wheel was removed, the cervical collar was applied. Then the cover of the chassis was removed to get full access to the seat. A strap system was used to completely immobilize the RD’s spine in the seat. Carrying the seat, the RD was extricated and carefully transferred onto a spine board.
5. Emergency extrication: Manual immobilization of the cervical spine was performed during the complete procedure. The seatbelt was opened and the RD moved forward as far as necessary to rotate him axially and pull him out of the car using the Rautek maneuver. Extrication ended when the RD was put onto a spine board in two meters distance.

**Fig. 4:**
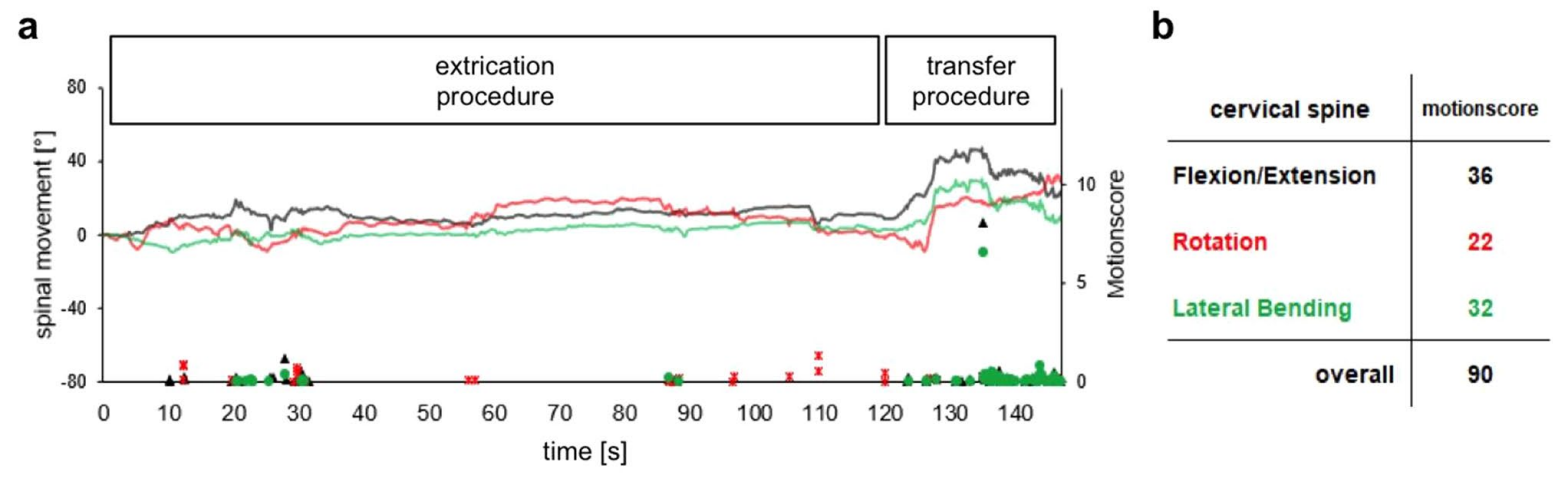
Cervical spine movement and resulting motion score during the extrication with the help of an extricable seat by the Extrication Team (**a**). Motionscore overview of the different ranges of motion of the cervical spine (**b**)

Due to the positioning of the Xsens unit at the forehead, the RD did not wear a helmet in any extrication scenario. As mentioned above, the professional Extrication Team underwent a special education and regular training on extrication procedures. However, the above-mentioned extrication procedures are not exclusively used in motorsport extrication. Urgent extrication with and without a rescue boa are also employed by emergency medical technicians (EMTs). Thus, these two procedures were also performed by a professional EMT team in order to compare the influence of special extrication training on the remaining spinal motion. All angular timely motion (Fig. 4a) was summed up to give a segmental motionscore (e.g. cervical spine motionscore; Fig. 4b). A mean segmental motionscore was calculated from the 3 runs performed (Table 1). All 4 mean segmental motionscores were summed up to a mean overall motionscore (Table 1).

**Table 1:** Summary of the mean motionscore and standard deviation (SD) of every spinal segment during the different extrication procedures performed by the Extrication Team (ET) and the emergency medical technicians team (EMT)

		Mean motionscore				
Procedure	Team	Overall ±SD	Cervical	Thoracic	thoraco-lumbar	Lumbar
Urgent extrication, no boa	EMT	**2932 ± 234**	1437	701	313	480
	ET	**2118 ± 644**	684	585	346	503
Urgent extrication with boa	EMT	**2725 ± 868**	758	936	382	649
	ET	**1589 ± 309**	198	735	266	389
Kendrick Extrication Device	ET	**2227 ± 223**	523	902	348	455
Extricable seat	ET	**1617 ± 526**	221	691	293	412
Emergency extrication	ET	**1448 ± 152**	254	500	217	477

### Statistics

Literature on the biomechanics of extrication procedures is rare. Thus, a sample size calculation based on previous experiments was not possible. It is unknown what amount of spinal motion is clinically relevant to cause secondary damage to a previously injured spine. We therefore decided to perform an explorative study without the requirement to present confirmatory statistic and statistical proofing study hypotheses. Data collection for the presented project is conducted using an electronic database system, specifically Microsoft Excel from Microsoft GmbH in Unterschleißheim/Germany. Detailed descriptive statistics are provided for all data collected. For continuous data and scores, the mean, standard deviation, 95% CI are calculated. Statistical analyses for mean, 95% CI and standard deviation (SD) were performed using the statistical software IBM SPSS software version 24.0.

Graphics were prepared using Microsoft Power Point 2011 and Adobe Photoshop CS4.

## Results

Rescue procedures in order to perform urgent extrication are performed in motorsports as well as in traffic accidents. In order to analyze the influence of special extrication training on the remaining spinal motion despite efforts to immobilize the spine, the two most frequently used procedures were performed by an Extrication Team as well as a professional EMT team.

With regard to the mean overall motionscore including all spinal segments, the Extrication team attained a motionscore of 2118 [95% CI 517–3718] and the EMT team a motionscore of 2932 [95% CI 1427–4435] (+ 38.4%) in case of urgent extrication *without* a rescue boa (Fig. 4). The higher mean overall motionscore by the EMT team was mainly due to a higher mean motionscore in the cervical spine 1437 (+ 110.1%) compared to the Extrication Team 684. The other spinal segments such as thoracic 585 vs. 701 (+ 19.8%), thoracolumbar 346 vs. 313 (-9.5%) or lumbar spine 503 vs. 480 (-4.6%) did not differ as much (Table 1).

When performing the urgent rescue procedure *with* the aid of a rescue boa, the EMT team achieved an overall mean motionscore in the same range 2725, [95% CI 568–4881] *with* vs. 2932 [95% CI 1427–4435] *without* boa. However, the mean overall motionscore was 71.5% higher compared to the Extrication Team 1589; [95% CI 819–2358] when using the rescue boa. When mean scores of individual spinal segments were analyzed, we found that the EMT team did especially worse in immobilizing the cervical spine 198 vs. 758 (+ 282.8%), but motion in the lumbar 389 vs. 649 (+ 66.8%) and the thoracolumbar spine 266 vs. 382 (+ 66.9%) was also increased.

**Fig. 5:**
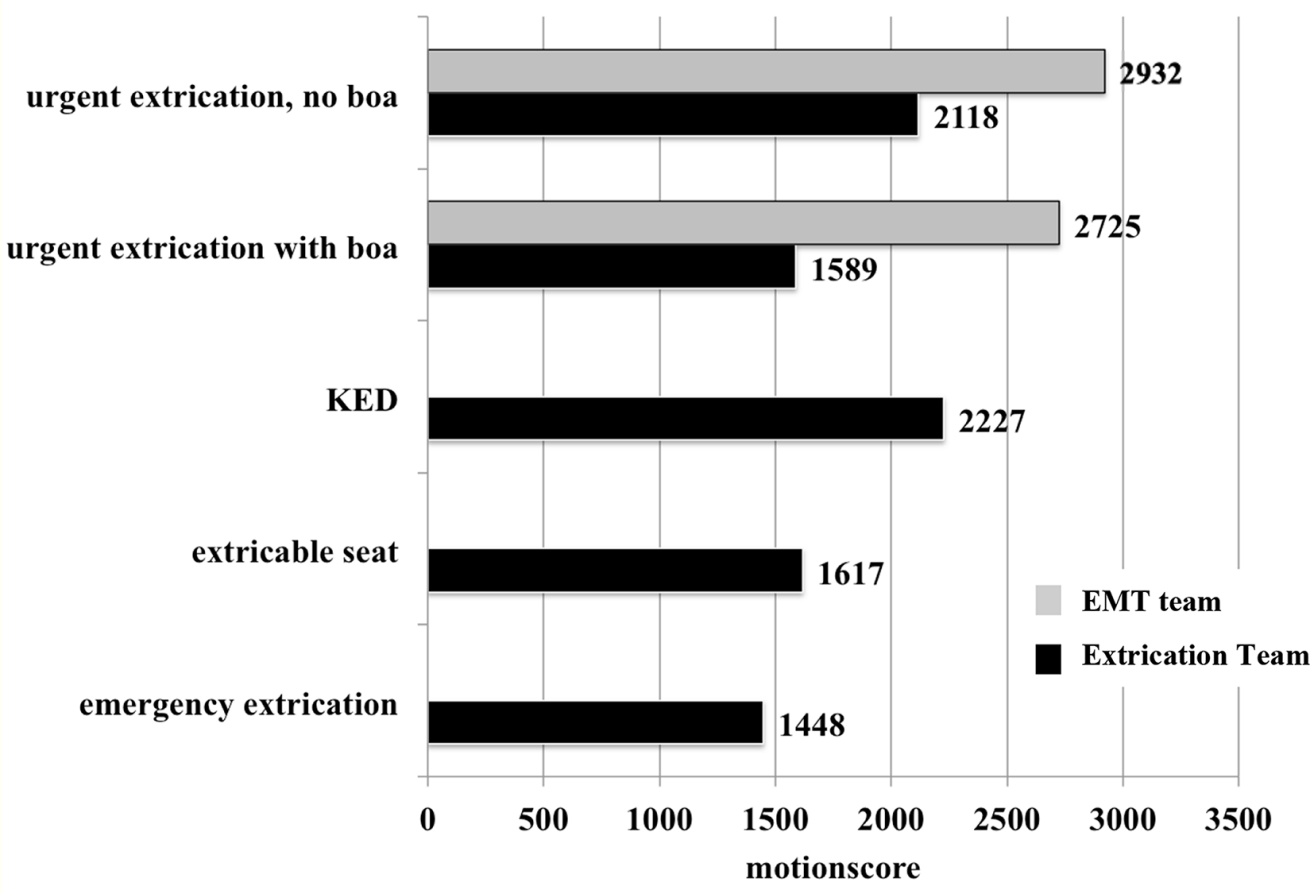
Comparison of mean overall motionscores of all extrication procedures performed by the Extrication Team and the emergency medical technicians (EMT) team; Kendrick Extrication Device (KED)

**Fig. 6:**
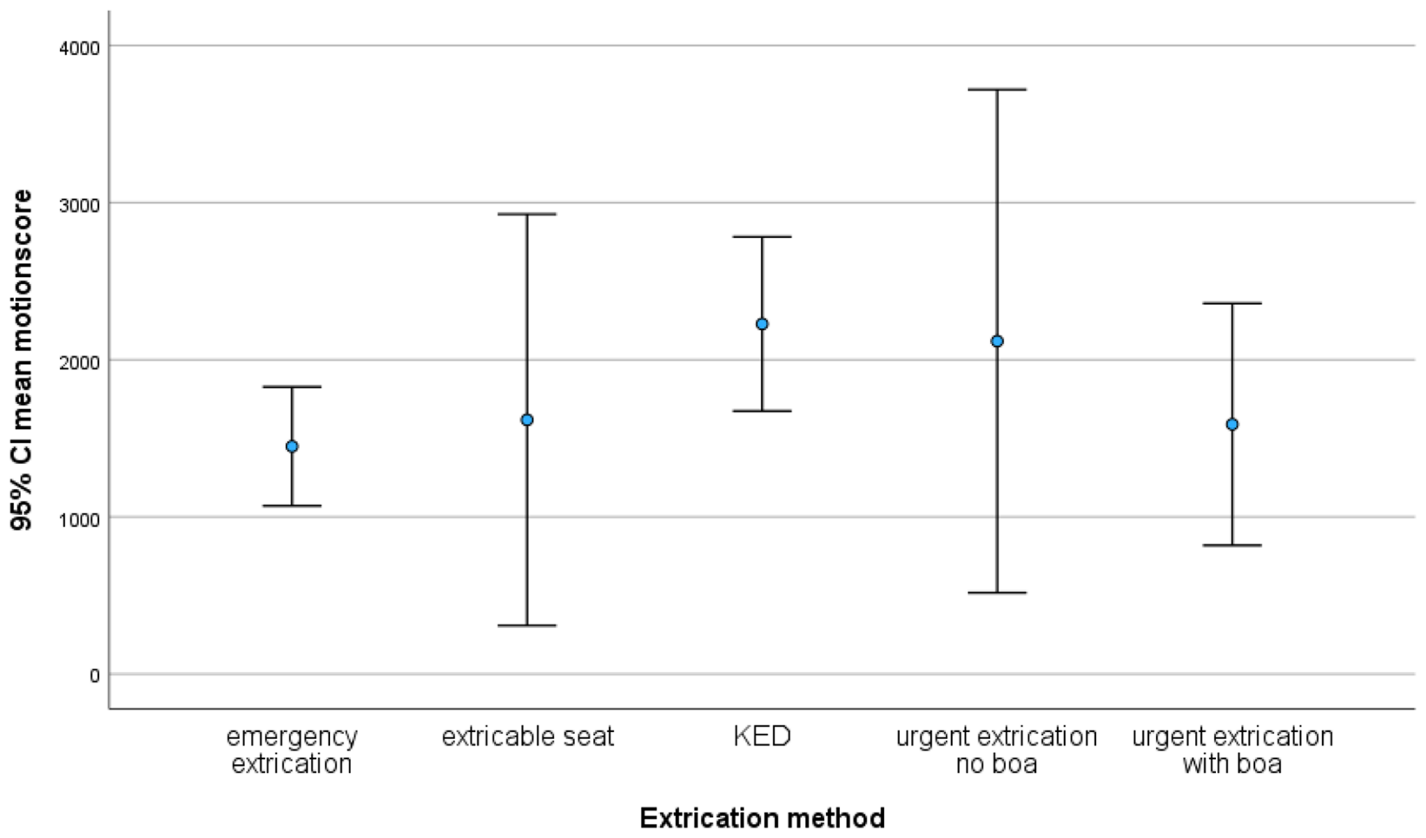
A comparison of the mean overall motionscore and 95% confidence interval (CI) of all extrication procedures performed exclusively by the extrication team. KED: Kendrick Extrication Device

Since some procedures (e.g. using the extricable seat; Fig. 1cd) require special training or devices (e.g. the KED; Fig. 2c), they were only performed by the Extrication Team. Using the rescue boa in case of urgent extrication reduced the mean overall motionscore from 2118 [95% CI 517–3718] to 1589 [95% CI 819–2358] (-25.0%; Fig. 5). The use of an extricable seat lead to a mean overall motionscore in the same range 1617 [95% CI 308–2926]; (Fig. 5), but the KED did not reduce mean overall motionscore 2227 [95% CI 1627–2782]; (Fig. 5) and was in the same range as the urgent extrication without a rescue boa 2118 [95% CI 517–3718]. The lowest overall motionscore was measured when performing an emergency extrication using not any device at all 1448 [95% CI 1070–1826] (Fig. 5). Details of all the techniques carried out by the ET team are shown in Fig. 6.

Spinal fractures resulting from motorsports accidents are commonly found in the cervical and thoracolumbar spine. Therefore, it is important to focus on these specific spinal segments when comparing the effectiveness of extrication procedures. Using a rescue boa, the mean cervical spine motionscore was lowest 198 (Table 1). The use of an extricable seat resulted in a mean cervical spine motionscore within the same range 221 (Table 1). However, even the emergency extrication– using not any device at all– resulted in an equivalent mean cervical spine motionscore 254 (Fig. 5). It markedly increased when the ET was using the KED 523 (+ 164.2%) or performing urgent extrication without a rescue boa 684 (+ 245.5%; Table 1). In the thoracolumbar segment, the mean motionscore was determined to be within a range from 217 up to 348 and therefore did not differ during different procedures (Table 1).

## Discussion

The study aims to evaluate extrication procedures from two different racecars, as there is a lack of data on this topic in the literature. Therefore we conducted a study on remaining spinal movement during various extrication procedures from racecars. Some of these procedures are also used following traffic accidents by EMT personnel on a daily basis and deserve to be examined with respect to their efficiency as their use remains controversial [20–23]. Beyond the racetrack, the World Health Organization attributes 50% of all spinal cord injuries to traffic accidents [24]. An estimated 3–25% of these injuries may be caused or aggravated by false immobilization procedures during transport or early inpatient treatment [21, 25]. Authors therefore agree that movement in all areas of the spine should be as minimal as possible [26–28]. Due to the demographic changes and increasing safety of automobiles, EMT missions to treat trauma patients make up only a small fraction and practicing extrication procedures has potentially been less focused on.

This study demonstrates that the professional Extrication Team achieved significantly better results than the EMT team in urgent extrication, with or without the use of a rescue boa. Our investigation of segmental spinal differences in immobilization efficiency revealed that the Extrication Team was particularly effective in protecting the cervical spine. This finding may be attributed to their well-founded knowledge of cervical spine injuries and regular training in cervical spine immobilization. Recent studies have highlighted the necessity of training EMTs in immobilizing the cervical spine [29, 30].

Both teams performed better when using the rescue boa. These results are supported by a recent study that showed less motion in the cervical spine when a rescue boa was used for urgent extrication from a civilian car [14]. In general, more rigid immobilization devices are thought to improve quality by allowing for better immobilization during procedures. However, our findings did not support this notion. The Extrication Team achieved their worst results when using the KED, despite being thoroughly trained in its use. Interestingly, the literature suggests that using fewer immobilization devices could result in less spinal movement during extrication procedures [14, 31]. Additionally, the Extrication Team successfully conducted an extrication on a RD using the extricable seat, which produced some of the best results in our analysis. The development of the extricable seat has substantially improved extrication from racecars, which is a technical engineering achievement [32].

The study found that the lowest mean overall motionscore was recorded during emergency extrication when using manual inline stabilization of the cervical spine and the Rautek extrication procedure. Despite being primarily used in acute danger to life of driver or emergency medicine personnel and having the least focus on spinal immobilization, the ET achieved their best results using this procedure. The crucial factor in achieving these results was the manual inline stabilization performed during the emergency extrication. Further studies are required to determine whether additional manual inline stabilization of the cervical spine could improve all other mentioned extrication procedures.

The findings of this study are not only pertinent to motorsport. The disparities observed between teams and procedures illustrate the significance of training and education in this field. In light of demographic trends, it can be postulated that the number of accidents involving elderly patients will increase. Comorbidities such as osteoporosis and other diseases can significantly impair bone and musculoskeletal stability, thereby rendering vehicle rescue techniques relevant to the entire population and civil road traffic.

The study has limitations, and caution should be exercised when interpreting the data. To the authors’ knowledge, there is no reliable data on the amount of strain (e.g. angular movement, axial force) that an injured spine can withstand. It was previously believed that spinal trauma could not be worsened by further manipulation [33]. However, recent cadaver studies have shown that standard emergency medical procedures directly affect the width of the dural sac in severely injured spines [7, 34]. Therefore, we have developed three axioms on which to base further analysis. The degree to which this theoretical approach accurately represents real, pathophysiological, and medically relevant exposure is uncertain. Therefore, this study does not provide absolute empirical evidence, but rather describes, with all its limitations, the movement of the spine during various release processes.

## Conclusions

Regular training of extrication procedures, as done by professional Extrication Teams in motorsports, paid off considerably reducing spinal movement during extrication from a crashed racecar. If available, extrication via an extricable seat should be performed. However, if an emergency extrication has to be performed for some reason, an additional manual cervical spine immobilization conducted with the Rautek maneuver can sufficiently reduce cervical spine movement.

## Data Availability

No datasets were generated or analysed during the current study.

## Code availability

Software application is available upon request to the corresponding author.
